# The impact of metabolic disorders on management of periodontal health in children

**DOI:** 10.1002/pdi3.38

**Published:** 2023-11-03

**Authors:** Yunyan Zhang, Tong‐Chuan He, Hongmei Zhang

**Affiliations:** ^1^ Chongqing Key Laboratory for Oral Diseases and Biomedical Sciences The Affiliated Hospital of Stomatology Chongqing Medical University Chongqing China; ^2^ Department of Pediatric Dentistry The Affiliated Hospital of Stomatology Chongqing Medical University Chongqing China; ^3^ Molecular Oncology Laboratory Department of Orthopaedic Surgery and Rehabilitation Medicine The University of Chicago Medical Center Chicago IL USA

**Keywords:** children, metabolic disorders, periodontal health

## Abstract

Periodontitis is a chronic inflammatory disease caused by plaque biofilm which shares risk factors with systemic chronic diseases such as diabetes, cardiovascular disease, and osteoporosis. Many studies have found increased prevalence and rate of progression of periodontal disease in children with common metabolic disorders. Although the causal relationship and specific mechanism between them has not been determined yet. The aim of this paper is to progress on the impact of metabolic disorders on periodontal health in children and the underlying mechanisms, which provides new evidences for the prevention and intervention of metabolic disorders and periodontitis in children.

## Graphical abstract

Metabolic diseases in children and adolescents may increase the risk of periodontal disease by affecting immunity and oral microbial colonization.

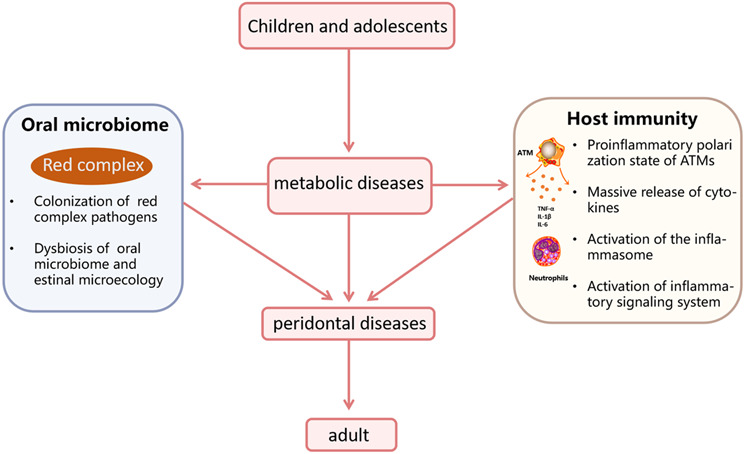

## 1 INTRODUCTION

Periodontal diseases are highly prevalent and can affect up to 90% of the worldwide population.^1^ Severe periodontitis can result in loosening of teeth, occasional pain and discomfort, impaired mastication, and eventual tooth loss. Not only that, periodontal diseases could even contribute to systemic inflammation. Recent studies have learned that not all individuals are at equal risk of developing periodontitis. Periodontitis with systemic diseases often has the characteristics of earlier onset, faster progression, more severe destruction, and worse response to treatment.^2^ A high prevalence of periodontitis is now found in adolescents and children with metabolic diseases such as obesity and diabetes.^3^, ^4^, ^5^, ^6^ Due to the particularities of children's growth and development, fewer studies have focused on the relationship between periodontal health and systemic diseases at this stage. However, preventive interventions in early life were found to be more beneficial than those in adulthood.^7^ Current epidemiological works found early disease experience could influence the risk of developing the same diseases in adulthood, especially metabolic diseases and chronic infections.^8^ The effects of these chronic inflammatory diseases on pediatric patients may also be long‐lasting. The aim of this review is to focus on the impact of common metabolic diseases on periodontal health in children, and to provide a theoretical basis for a better understanding of the potential mechanism of systemic metabolic diseases on periodontal disease.

## 2 DEVELOPMENT OF PERIODONTAL TISSUES IN CHILDREN AND ADOLESCENTS

Compared with adult periodontal health, children's periodontal health receives less attention. Due to the complexity of growth and development in childhood and adolescence (i.e., the structural and functional changes of periodontal structure during the eruption and exfoliation of teeth,^9^, ^10^, ^11^, ^12^ the establishment and maturation of oral microflora,^13^, ^14^, ^15^, ^16^ and the gradual development of the immune defense system^17^), which means more attention should be paid to distinguishing the pathological process and normal changes of periodontal tissue with age.

Periodontal tissue consists of periodontal ligament, cementum, and alveolar bone. Typically, children will go through primary dentition, mixed dentition, and young permanent dentition during the growth and development. Each stage has its own characteristic physiological condition of periodontal tissue. Periodontal tissue in deciduous dentition has several characteristics: 1. The color of gingiva is pale red for rich blood vessels (the color will become lighter with age). The epithelium is thin, the degree of keratosis is poor, and the connective tissue of the lamina propria is loose.^18^ 2. The free gingiva of deciduous teeth is slightly thicker than that of adults, with rounded edges. The width of the attached gingiva is wider in adults than in children.^19^ 3. The periodontal ligament is wider and the fiber density is lower than that of adults. The alveolar bone has less bone trabecula and calcification, more bone marrow space, and more blood supply and lymphatic drainage.^20^, ^21^

Due to the changes of hormone levels, the replacement of primary and permanent teeth and the rapid growth of maxillofacial bone in mixed dentition, there are diverse characteristics and individual differences: 1. Gingival may have a darker appearance^22^ 2. Root migration of junctional epithelium. It is generally believed that the distance between the cementum enamel junction and the alveolar bone crest (CEJ‐ABC) is 1–2 mm in the deciduous stage, indicating that the height of alveolar bone is normal.^18^ When the alveolar bone is adjacent to the replaced primary teeth or erupted permanent teeth, the distance of CEJ‐ABC greater than 2 mm is considered physiological. In the study of deciduous teeth in vitro, it was found that 94% of deciduous molars had attachment loss, with an average value of 0.26 ± 0.32 mm.^22^ This tiny loss of attachment has no clinical significance and does not necessarily prove the existence of periodontitis, as it may be physiological. Many scholars have their own understanding of this loss of attachment, which may be related to inflammation, marginal periodontitis, or tooth eruption. The apical migration of the junctional epithelium has been related to the physiological apical displacement of the dento‐gingival junction during the eruption of human permanent teeth and the increase in the distance of CEJ‐ABC.^22^ A non‐linear increase in the distance from the CEJ to the ABC takes place with age, this phenomenon may be related to facial growth patterns.^23^ During facial growth, the maxilla and mandible are displaced in an anterior and inferior directions (primary displacement), a “space” is created and alveolar bone remodeling takes place with a consequent vertical drift of the teeth.^24^ Tooth eruption may take place at a faster rate than the ABC deposition. Another factor that should be considered is that the root migration of junction epithelium may be related to the loss of adjacent primary teeth or the eruption of permanent teeth.^25^ However, in most cases, the 2 mm CEJ‐ABC distance may be considered as the boundary of “healthy” alveolar bone height. In conclusion, due to the special nature of dental replacement in children and adolescents during growth and development, periodontitis cannot be defined as precisely as it is in adulthood. Therefore, the periodic examination of periodontal tissues is critical for children.

## 3 PREVALENCE, DIAGNOSIS AND CLASSIFICATION OF CHILDREN'S PERIODONTAL DISEASES

Periodontal disease generally refers to chronic inflammation of periodontal supporting tissues including gingiva, periodontal ligament, and alveolar bone.^26^ Periodontal diseases are recognized nowadays as epidemics in children, adolescents, and adults, though they were often considered in the past to be a disease associated with aging. Periodontal health problems in children have not received as much attention as adult periodontal diseases. However, there is less evidence in prospective long‐term studies that periodontitis symptoms in childhood strongly predict the risk of periodontal disease in adulthood. In a longitudinal study spanning 32 years, people with high levels of dental plaque as children and adolescents experienced the highest levels of dental caries and periodontal disease as adults aged.^27^ A study of adolescents with severe dental caries in early childhood found that they were more than four times more likely to develop dental caries than age matched controls. They have a higher prevalence of periodontitis, more likely to be overweight or obese, and have a poorer quality of life related to oral health.^28^

The classification system of periodontitis agreed at the 1999 International Workshop is generally used at present. It includes gingival diseases, chronic periodontitis, aggressive periodontitis, periodontitis related to systemic diseases, necrotizing periodontal diseases, abscesses of the periodontium, and periodontitis associated with endodontic lesions.^29^ The basic periodontal examination has been advocated to screen for periodontal diseases in adults. According to the American Academy of Pediatrics and Dentistry,^30^ children and adolescents should have periodic periodontal assessments and records. It includes the community periodontal index and X‐ray film observing the shape of alveolar bone edge for identifying early signs of periodontal destruction. The definition of periodontitis cases depends to a large extent on the disease degree (the number of affected teeth) and the specific threshold of the disease severity (the depth of periodontal pockets of affected teeth, degree of attachment loss and alveolar bone loss). Thus, the estimation of the prevalence of periodontitis in different populations is essentially different. The prevalence of periodontitis in children and adolescents ranges from 2.2% to 80%. In the majority of the population, more than 70% of children over 7 years old suffer from periodontitis.^31^, ^32^ Loss of periodontal attachment and supporting bone can be found at one or more sites in 5%–9% of children aged 5–11 years and in 5%–46% of children aged 12–15 years.^33^ A study found that the prevalence of gingivitis among American teenagers reached 82.1%.^34^ Other studies around the world have reported similar results, with the high prevalence of periodontitis in children and adolescents.^35^, ^36^ According to the epidemiological data, periodontal diseases in children and adolescents are mainly characterized by mild and chronic periodontitis, but at this stage, severe periodontal diseases involving the entire dentition can also occur, and most of the indications are related to systemic diseases. Next, we will summarize the common metabolic diseases in children related to periodontitis.

## 4 COMMON METABOLIC DISORDERS AND PERIODONTAL MANIFESTATIONS IN CHILDREN

Periodontitis, as a chronic inflammation, is epidemiologically linked to many chronic inflammation driven diseases.^37^, ^38^ In recent years, there has been a growing body of research on periodontitis and systemic health. The systemic oral health connection is not only the result of common risk factors, but is driven in large part by a variety of microbe‐induced immune mechanisms.^39^, ^40^ Host immune responses are tightly intertwined with metabolism, and dysfunction of this integrated system may contribute to chronic metabolic inflammatory diseases such as obesity, metabolic syndrome (MetS), and type 2 diabetes mellitus (T2DM).^40^ Chronic low‐grade inflammation is a unifying feature and contributing factor to these diseases. Thus, at least in principle, metabolic diseases may affect periodontal inflammatory conditions by increasing the systemic inflammatory burden.^41^, ^42^ At the same time, periodontal diseases are a common manifestation of some systemic diseases and may have important diagnostic value and therapeutic implications.

### 4.1 Obesity

Obesity is a chronic metabolic disease, which will lead to a systemic inflammatory state and insulin resistance. Obesity increases the risk of many chronic diseases, including hypertension, dyslipidemia, diabetes, cardiovascular disease and osteoarthritis, with a significant impact on children's physical and mental health. Overweight and obesity among children and adolescents have become one of the most serious global public health concerns in the 21st century. In the past 30 years, the global prevalence of childhood obesity has increased significantly.^43^ A study systematically estimated the prevalence of overweight and obesity among children (<20 years old) and adults in 195 countries from 1980 to 2015. It found that since 1980, the childhood obesity rate in more than 70 countries has doubled, and that in some developing countries, the childhood obesity rate has roughly tripled. In many countries, the growth rate of childhood obesity has been higher than that of adult obesity.^44^ In China, the latest national prevalence estimates for 2015–2019 were 6.8% for overweight and 3.6% for obesity in children younger than 6 years, 11.1% for overweight and 7.9% for obesity in children and adolescents aged 6–17 years, nearly double compared with 2005.^45^ Reeves at al. found that a 1‐kg increase in body weight may increase the risk of periodontal disease by 6% in the group of obese teenagers over 17.^46^ A cross‐sectional study found a significant increase in the values of periodontal disease indicators and a higher percentage of pockets with a probing depth exceeding 4 mm in obese children aged 6–13 compared to peers with normal body weight.^47^ Moreover, an increased expression of TNF‐α existed in gingival crevicular fluid samples from the obese children.^48^, ^49^ In conclusion, obese children and adolescents are at a higher risk of periodontal disease.

### 4.2 Diabetes mellitus

Diabetes can be divided into type I and type II diabetes. Type 1 diabetes mellitus (T1DM) is a type of diabetes caused by destruction of pancreatic beta cells and absolute insulin insufficiency. It usually starts in adolescence and presents as a severe disease state with ketoacidosis. Type 2 diabetes is a type of diabetes mainly caused by insulin resistance or accompanied by insufficient insulin secretion for various reasons, accounting for more than 90% of diabetes patients. The patients were characterized by hyperglycemia, relative insulin deficiency, insulin resistance, etc. Type 1 diabetes is still the most common metabolic disease in children. The incidence rate of type 1 diabetes increases with age, reaching its peak around 10–14 years old, but this disease can occur at any age.^50^ Globally, the incidence rate of type 1 diabetes began to increase in the 1950s, with an average annual increase of 3%–4% in the past 30 years.^51^, ^52^, ^53^

An increasing number of children, adolescents and young adults are being diagnosed with type 2 diabetes. In the United States, the prevalence of T2 DM among children and adolescents increased by 30.5% between 2001 and 2009.^54^ Data from China suggest that the prevalence of type 2 diabetes in children has increased dramatically over the past 20 years.^55^ Several studies have shown that diabetes (types 1 and 2) is an established risk factor for periodontitis and contributes to the increased prevalence, severity and progression of periodontitis. Importantly, accumulating epidemiological evidence suggests a positive association between obesity^56^, ^57^, ^58^ and MetS^59^, ^60^, ^61^ (both diseases are strongly associated with type 2 diabetes) and periodontitis. Children with diabetes had significantly more gingival inflammation and attachment loss than control children.^62^ The incidence of chronic gingivitis in patients with type 1 diabetes is significantly higher than that in the healthy population.^63^ Gingival index (GI) refers to a comprehensive evaluation of the degree of inflammation of the gums based on their color, shape, texture, and probing bleeding. GI was significantly higher in obese children with T2DM than in obese children without diabetes and children with normal body weight aged.^64^ A cohort study of 350 children aged 6–18‐year‐old found a strong positive association between mean hemoglobin A1c and periodontitis.^65^ These studies suggest that both type I diabetes and type II diabetes increase the risk of periodontal disease in children and adolescents.

### 4.3 Metabolic syndrome

The “MetS” is a complex syndrome of metabolic disorders caused by overnutrition, sedentary lifestyle and obesity. Mets comprises a clustering of abdominal obesity, insulin resistance, dyslipidemia, and elevated blood pressure. Mets is associated with other comorbidities, including a prothrombotic state,a proinflammatory state and nonalcoholic fatty liver disease (NAFLD).^66^ Since MetS is a cluster of different conditions, rather than a single disease, leading to the development of multiple concurrent definitions. Although there is no international common definition of the MetS in children and adolescents, all definitions include obesity as a prerequisite for the development of the MetS even in children. Obesity is one of the major cardiometabolic risk factors, which is closely related to other metabolic diseases such as hyperlipidemia, hyperinsulinemia, and hypertension. A consensus definition was published in 2007 by the International Diabetes Federation, which agreed that 10‐year‐old children met the criteria for MetS if they had at least three of the following risk factors: high waist circumference, hypertension, insulin resistance, and dyslipidemia.^67^ A Spanish cross‐sectional study published in 2011 showed that Mets occurred in 8%–32% of prepubertal children and 9.7%–41.2% of adolescent children.^68^ Furthermore, Reinehr et al. compared different definitions of MetS in a cohort of 1205 children and found a wide prevalence range from 6% to 39%.^69^ Notably, MetS increases the risk of development and progression of periodontitis.^70^ Boys diagnosed with MetS had significantly higher levels of gingival crevicular fluid tumor necrosis factor alpha (TNF‐α) and more sites with gingival bleeding compared with healthy boys.^71^ What's more, the number of positive MetS parameters, and HDL‐cholesterol parameter showed a significant association with gingivitis in adolescents. Adolescents with a larger number of positive MetS parameters, and low HDL‐cholesterol level were likely to have gingivitis.^72^

### 4.4 Metabolic bone disease

#### 4.4.1 Pediatric rickets

Pediatric rickets, characterized by bone‐deformities, is due to defective mineralization and disruption of chondrocyte maturation in growing bones.^73^, ^74^ Pediatric rickets caused by Vitamin D insufficiency and disorders of calcium and phosphorus metabolism is a major public health problem worldwide, with the reported prevalence of up to 70% in some developing countries.^75^ The Rochester Epidemiology Project reported that the incidence of rickets has been increasing substantially over the past 40 years (0, 2.2, 3.7, and 24.1 per 100,000 in the 1970s, 1980s, 1990s, and 2000s, respectively).^76^

#### 4.4.2 Osteogenesis imperfecta, Ehlers Danlos syndrome, and Marfan's syndrome

Hypophosphatemic chondropathy is a disorder of bone mineralization caused by excessive urinary phosphorus excretion.^77^ X‐linked hypophosphatemic chondropathy is the most common cause of hereditary chondropathy, affecting approximately 1 in 20,000 live births.^78^, ^79^, ^80^ Other less common causes of hereditary hypophosphatemia include autosomal dominant hypophosphatemia, autosomal recessive hypophosphatemia and hereditary hypophosphatemic chondropathy with hypercalciuria. Acquired hypophosphatemic chondropathy can be caused by tumor induced osteomalacia and Fanconi syndrome.^77^ Congenital X‐linked hypophosphatemic rickets have more pronounced systemic symptoms and oral manifestations with marked skeletal and dental mineralization disorders.^81^, ^82^ Baroncelli at al. found children with X‐linked hypophosphatemic rickets had increased incidence of periodontal disease.^82^

Osteogenesis imperfecta, also known as brittle bone disease, is the most common inherited bone disorder with an incidence of 0.79 per 10,000 newborns.^83^ Osteogenesis imperfecta is a heterogeneous group of inherited connective tissue disorders associated with abnormal type I collagen leading to a variety of clinical manifestations.^84^, ^85^ There are four categories of osteogenic defects. Type I; Mild phenotype, type II; Perinatal fatal, type III; Progressive deformity, most severe surviving form, type IV; Intermediate severity between types I and III.^86^ EDs are a group of inherited connective tissue disorders caused by collagen and elastin, leading to a variety of disorders. There are 13 types, many of which are associated with increased skeletal and capillary fragility in children and adults.^87^ MFS is an autosomal dominant connective tissue disorder with a reported incidence of 1 in 3000–5000 individuals.^88^ MFS has a wide range of clinical manifestations, including cardiovascular, musculoskeletal, cutaneous, and central nervous systems.

### 4.5 Non‐alcoholic fatty liver

NAFLD is the most common cause of chronic liver disease in western countries.^89^, ^90^ In particular, there is an alarming increase in the number of children affected by NAFLD, which is supported by high prevalence data, ranging from 3% to 12% in the general pediatric population and up to 70%–90% in young obese individuals.^91^ Childhood NAFLD is associated with several factors of MetS, such as abdominal (central) obesity, dyslipidemia (hypertriglyceridemia and/or hypercholesterolemia), and insulin resistance.^92^ Thus, NAFLD can be considered a hepatic manifestation of MetS. The NAFLD shows a significant association with clinical microbial periodontal parameters.^93^ A similar association was observed between periodontal disease and NAFLD risk (OR = 1.19, 95% CI = 1.06–1.33).^46^

## 5 THE MECHANISM OF METABOLIC DISEASES AFFECTING PERIODONTAL HEALTH MANAGEMENT IN CHILDREN

The identification of etiology is critical for the prevention and treatment of periodontal diseases. However, the specific etiological mechanisms of periodontal disease have not been fully understood. Currently, the recognized etiologies are dysbiosis of oral microbiome and dysregulated host immune response.^40^ Metabolic diseases can not only affect the oral and intestinal microbiota balance, but also destroy the host immune function. A poorly controlled host immune response, in turn, can generate a self‐perpetuating pathogenic cycle where dysbiosis and inflammation reinforce each other by forming a positive feedback loop.^94^

### 5.1 Effects of metabolic disorders on host immunity

**FIGURE 1:**
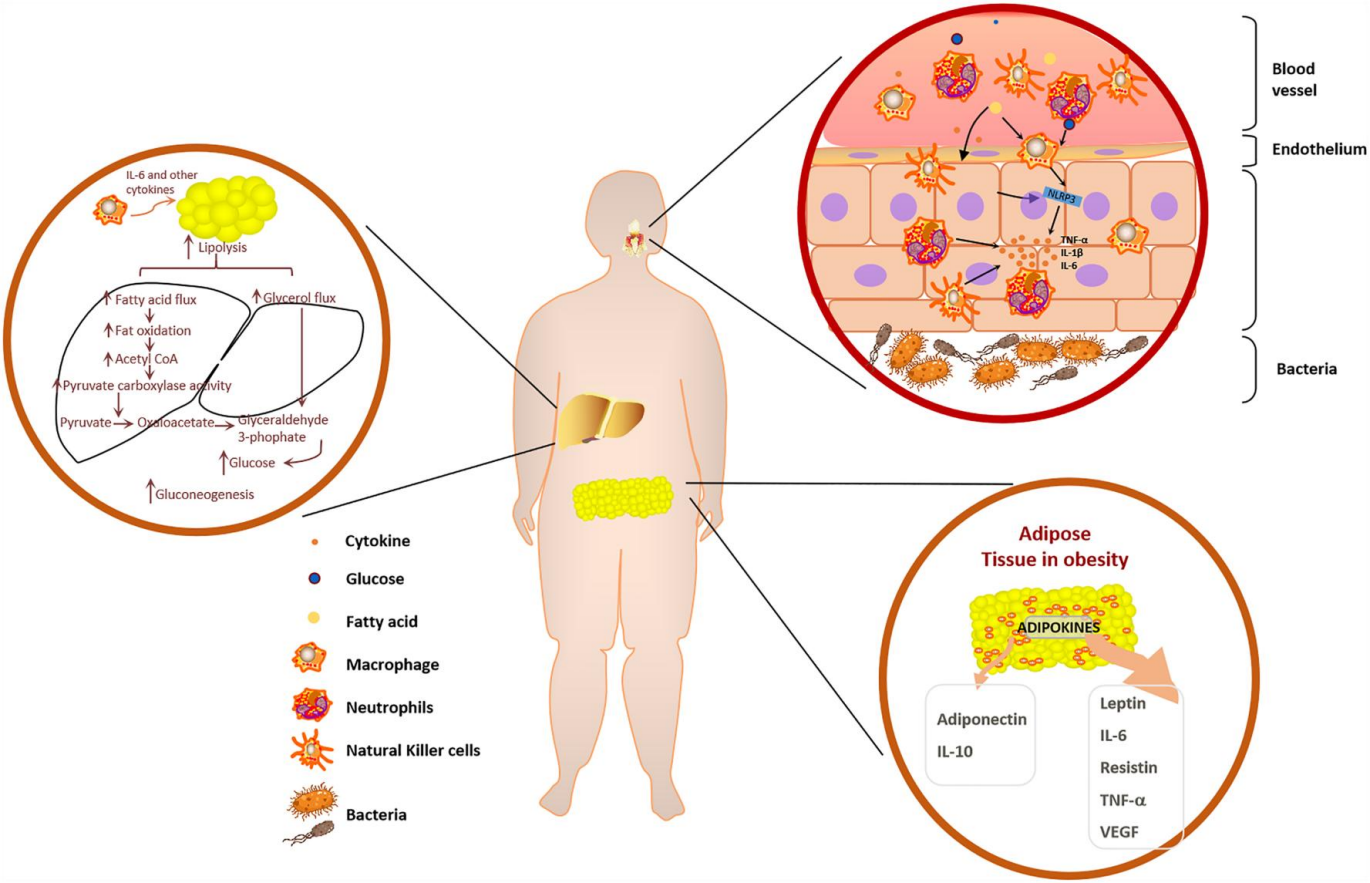
The influence of disorders in lipid and glucose metabolism on periodontal inflammation. Leptin and pro‐inflammatory cytokines are released from adipose tissue in obesity to recruit and activate pro‐inflammatory immune cells. These pro‐inflammatory cytokines and immune cells will follow the blood into metabolic organs, such as the liver, causing chronic inflammation and affecting the process of glucose and lipid metabolism. Glucose and free fatty acids in the blood, among others, activate the inflammasome, which releases IL‐1β, creating a highly pro‐inflammatory environment in periodontium. IL‐1 β, interleukin‐1β.

As previously mentioned, common metabolic diseases in children are most frequently associated with abnormal glucose and lipid metabolism. Therefore, we mainly focused on the effects of obesity and diabetes on host immunity here. Pediatric obesity has both short‐term and long‐term impacts as the physiological changes altered by obesity occur at critical developmental stages. These comorbidities are caused by obesity‐related low‐grade inflammation, characterized by abnormal cytokine production and macrophage infiltration into adipose tissue.^95^ Large differences in leukocyte numbers as well as in phagocytic and oxidative burst activity of monocytes, have been reported between normal and obese individuals (Figure 1).^96^ Not only that, insulin target tissues such as adipose tissue, liver, muscle, and pancreatic islets are under attack from chronic inflammation in children with obesity and diabetes.^97^, ^98^ Adipose tissue‐associated inflammation elicits a wide variety of immune responses, involving early neutrophil participation followed by macrophage involvement and mast cell polarization.^96^ These cellular adaptations lead to altered metabolic profiles in early life and premature death in adulthood.^99^ Studying the causes of obesity‐related inflammation in pediatric populations may identify opportunities to prevent progression to serious comorbidities such as hypertension, abnormal glucose metabolism, and dyslipidemia. These observations have led to the term “immunometabolism,” which encompasses the potential interplay between immune processes and metabolic defects.^100^ In the context of pediatric obesity, adipose tissue displays unhealthy expansion, with excessive accumulation of adipocytes, leading to hypoxic conditions in this tissue.^101^ The hypoxic environment in turn triggers the recruitment of monocytes that subsequently convert to mature adipose tissue macrophages (ATMs).^102^ Proinflammatory macrophage recruitment, accumulation, and activation in metabolic tissues are the ultimate drivers of chronic low‐grade inflammation. Although macrophages are the major effector cell type, other types of immune cells also participate in these inflammatory processes.^103^, ^104^ The proinflammatory polarization state of ATMs leads to the release of a large number of inflammatory cytokines. Additionally, macrophages are capable of secreting chemotactic molecules such as TNF‐α.^103^ In a cohort study of obese Mexican American children, alterations in blood plasma cytokines/chemokine levels among healthy weight, overweight, and obese children were found.^105^ Serum concentrations of interleukin‐8 and TNF‐a were higher in the obese children. The release of TNF‐a not only recruits other inflammatory factors involving interleukin‐1β (IL‐1β) and interleukin‐6 (IL‐6)^106^ but also can activate various intracellular signaling molecules, such as JNK and IKKB, which are key components of the inflammatory signaling system, leading to impaired insulin action.^107^ The adipose tissue of obese children expresses high levels of TNF‐α and its inhibitor can improve insulin sensitivity and glucose tolerance abnormalities which are vital findings in establishing the link between immune cells and metabolic dysfunction.^104^ In a rodent model of obesity, normalization of TNF‐α decreased insulin resistance.^104^ Another key component of inflammation activation is a multimeric protein complex called the “inflammasome,” which is activated by cellular nutrients such as glucose and free fatty acids to induce IL‐1β production (Figure 1).^107^, ^108^ Interestingly, obesity has been demonstrated to exacerbate thymic senescence, reducing T‐cell diversity, and therefore potentially affecting immune surveillance.^109^

On the other hand, obesity has a variety of impacts on adipose cells, particularly the endocrine effects of adipokines (Figure 1). The main immunomodulatory factors derived from adipose include leptin, adiponectin, and proinflammatory cytokines: TNF‐α, IL‐6, and IL‐1β.^110^, ^111^ The levels of adiponectin, declined during obesity, have been shown to affect natural killer cell cytotoxicity and cytokine production.^112^ At the same time, excessive proinflammatory cytokines produced by the white adipose tissue of obese individuals can be secreted into the blood and may have long‐term effects. However, how the long‐term production of these cytokines affects cellular immunity remains to be elucidated. Prolonged exposure to proinflammatory cytokines may decrease the sensitivity of immune cells to inflammatory responses during actual infections.^113^ Compared to proinflammatory cytokines, the pleiotropic effects of leptin on immune cell activity are highly diverse and complex.^114^ Almost all cells of the innate immune system express an isoform of the leptin receptor OBRb, which is required for leptin signaling.^115^ In monocytes, leptin upregulates the production of the proinflammatory cytokines IL‐6, IL‐12, and TNF‐α, as well as phagocytic function.^116^ In neutrophils from healthy humans, leptin signaling induces chemotaxis, production of reactive oxygen species, and affects oxidative capacity.^117^ Natural killer cells are greatly influenced by leptin signaling, including differentiation, proliferation, and activation.^118^ Ob/ob mice, an animal model of obesity, provided important information about the role of leptin in host defense and immunity, with nearly all innate immune cells being impaired in mice lacking intact leptin signaling.^119^ In conclusion, the clinical observations and the results of animal experiments suggest that obesity impairs the normal functioning of the immune system.

Similar to obesity, diabetes also leads to hyperreactivity of the body's immune system and immune cells. T1DM is a long‐term and chronic autoimmune disorder, in which the immune system attacks the pancreatic cells.^120^ Early evidence in rats and humans suggested a defective neutrophil response in diabetes.^121^ Subsequent studies have shown that diabetic patients develop a hyperinflammatory, monocytic phenotype characterized by elevated levels of proinflammatory mediators in the periodontal sulcus fluid.^122^ A number of experimental and clinical data have clearly established that adipose tissue, liver, muscle, and pancreas are sites of inflammation in the presence of obesity and T2DM.^123^, ^124^, ^125^ An infiltration of macrophages into these tissues is seen in animal models of obesity and diabetes as well as in obese human individuals with MetS or T2DM.^126^, ^127^ Therefore, diabetes may increase the local (infected site) and systemic inflammatory response to bacteria.^128^ Moreover, the production of IL‐1 and TNF‐α was raised in diabetes by increasing the polarization of M1 macrophages, which may exacerbate periodontal disease.^129^ Dendritic cells regulate adaptive immune response by activating lymphocytes. Diabetes may potentially affect dendritic cells, increasing the production of Th1 or Th17 lymphocytes or reducing the formation of regulatory T cells.^128^ Therefore, the role of the immune inflammatory response in the pathogenesis and relationship between periodontitis and metabolic disorders cannot be ignored. The chronic inflammatory environment caused by metabolic disorders not only increases the susceptibility of periodontitis, but also affects the regeneration of periodontal tissues. Regulation based on immune response is a hot topic and research direction in the prevention and treatment of periodontitis and metabolic disorders.

### 5.2 Disrupted equilibrium of the oral microbiome in metabolic disorders

The oral cavity is the second largest microbiota in the human body. Currently about 500 different bacteria have been identified in the oral cavity.^129^ The microbiome and the host's immune system are interdependent and co‐develop from birth. Thus, the microbiota shapes the development of the immune system, which in turn determines the composition of the microbiota. The composition of the oral microbiota varies according to different life events: dietary diversification, hormonal changes (puberty and menstrual period), administration of drugs such as antibiotics, and age.^130^ Another source of alterations in the oral microbiota is changes in the balance of systemic health, generally associated with systemic diseases such as metabolic disorders.^131^

Levels of several bacteria are higher in the oral cavity of obese individuals than in nonobese controls, and these bacterial species appear to serve as biological indicators for the development of overweight conditions. Obesity is linked to subgingival microbiota disturbance in adolescents. Previous study has demonstrated that traditional periodontal pathogens such as *Porphyromonas gingivalis*, *A. actinomycetemcomitans*, and *P. micra*,^132^, ^133^ are present a threefold increase in the dental biofilm of obese adolescents compared with the normal weight controls. A significantly higher number of bacteria from the *Streptococcus* genus were found in the group of children with well‐controlled diabetes mellitus when compared to the healthy children.^134^ Glycemic control of childhood type I diabetes was also associated with the complexity and abundance of microbial communities in dental plaque, as discovered by 16S rRNA sequencing. Besides, several studies have reported diabetes induced changes in the oral microbiota. Examples of these bacterial changes include: (1) increased phagocyte function in patients with diabetes^135^; and (2) *P. gingivalis*, *Tannerella forsythia*,^136^, ^137^ Capnocytophaga, *Pseudomonas*, bergeria, *Sphingomonas*, *Corynebacterium*, *Propionibacterium*, and *Neisseria* were increased in hyperglycemic individuals.^138^ Compared with the healthy population, the salivary bacterial spectrum of MetS patients has changed with decreased diversity of bacterial species.^139^ When further stratified, both the MetS healthy periodontium and MetS periodontitis subject groups exhibited relatively distinct microbial profiles from each other and from those of healthy subjects. In addition, the MetS periodontitis group showed greater enrichment of canonical periodontal “Red complex” pathogens, namely forsythia, spiral hilum, and *Treponema*. However, for *P. gingivalis*, no significant difference was detected between the MetS patients and healthy people.^140^ Periodontopathic bacteria, particularly *P. gingivalis*, have been associated with the pathogenesis and progression of NAFLD on the basis of clinical research and immunology.^141^ The oral derived bacterium *P. gingivalis* can be detected in both liver and feces of patients with NAFLD. Studies have identified a possible oral‐gut‐liver axis in NAFLD patients.^142^, ^143^ Similarly, an American study showed the transfer of oral bacteria into atherosclerotic plaques.^144^ Furthermore, they described the potential role of oral microbiota biomarkers in the development of vascular diseases.^144^, ^145^ By sequencing the oral microbiota, they identified a positive correlation between the abundance of gram‐negative oral bacteria and the blood levels of total cholesterol and low density lipoprotein in patients with atherosclerosis.^146^, ^147^, ^148^ It suggests translocation of oral bacteria into the systemic circulation.

The close interaction between oral microbes and metabolic diseases have also been found in animal experiments. Oral microflora of diabetic mice was transferred from diabetic hosts to germ‐free mice and compared with bacteria transferred from normoglycemic mice to similar hosts. Compared with bacteria from the normoglycemic control group, the transfer from the hyperglycemic mice stimulated more infiltration of neutrophils and the expression of bone resorbing cytokines (IL‐6 and RANKL), increasing the number of osteoclasts and more periodontal bone loss.^149^ Moreover, inoculation of *P. gingivalis* to the mouth of ApoE‐deficient mice caused an increase in the number of *Bacteroides* and a decrease in the number of Scleroderma, which were closely related to endotoxemia and systemic inflammatory responses.^150^ Many clinical studies have found that the development of atherosclerosis can be suppressed correspondingly after periodontitis is controlled in patients.^151^, ^152^ By inducing obese mice with a high‐fat diet, the researchers found that obese mice developed alveolar bone resorption and alterations in oral microbiota similar to those induced by actinomycetes. Obesity‐induced alveolar bone resorption was effectively improved by oral topical application of antibacterial agents in mice with a high‐fat diet.^153^

Metabolic diseases can not only affect the colonization of oral microbiota, but also break the balance of intestinal microecology, which is closely related to the development of children's immune system.^154^ The dysbiosis of oral microbiome cannot be improved solely through antibiotic treatment, but requires adjustments to the systemic immune system. In conclusion, metabolic diseases affect the whole‐body microbiome to a certain extent. Such changes may break the balance between the host immunity and the microbiome, causing chronic inflammation and aggravating periodontitis. This implicates a guiding direction for future research on the treatment of metabolic diseases complicated with periodontitis, but it still needs further research.

## 6 CONCLUSION AND FUTURE DIRECTIONS

As mentioned before, systemic metabolic diseases are closely related to the periodontal health of children and adolescents, giving some advice to the clinicians in the treatment. For physicians, enough attention should be paid to whether the patient has periodontal diseases when treating systemic metabolic diseases. The risk of systemic disease should be considered for oral practitioners in periodontitis patients with poor therapeutic effect of topical treatment. In conclusion, concurrent therapy with control of infection and inflammation should be used in adolescents and children with both metabolic and periodontal conditions. For example, screening for HbA1c is recommended at the time of the children's oral examination, with the aim of selecting patients at high risk for periodontitis and tooth loss or for incidental findings of an underlying cause of disease. There is some evidence that showed that nonsurgical treatment of periodontitis did improve glycemic control in patients with diabetes.^155^, ^156^, ^157^ The study found that non‐surgical periodontal treatment resulted in a 0.24%–1.21% point decrease in glycated hemoglobin after 3 months of intervention.^158^ However, the effect of hypoglycemic agents on periodontal status remains unclear. Studies have shown that only in animal experiments can the application of rosiglitazone improve tissue damage associated with periodontitis in diabetic rats.^159^, ^160^ The effect of the diabetes drugs on periodontitis may mainly depend on its anti‐inflammatory potential, rather than the reduction of glycosylated hemoglobin.

Certainly, based on epidemiological data, the incidence of periodontal disease is not as high in children or adolescents as it is in adults. Due to the age‐dependent response of gingival tissue to oral bacteria, infants and young children tend to exhibit diminished clinical signs of gingival inflammation, even in the presence of a substantial microbial, especially if the severity of gingival inflammation does not appear to be proportional to plaque accumulation.^161^, ^162^, ^163^ More importantly, the available literature does not strongly support the idea that the periodontal pathogens causing periodontitis in adults have their onset later in life. Indeed, these potential pathogens appear to be acquired early in life and at low levels in the oral microbiome of children and adolescents.^164^ Once an individual's native oral microbial ecology is established, it seems difficult for alien bacteria to gain a foothold for permanent colonization. What seems to occur is that changes in the oral environment contribute to the selection of the emergence of various taxa, genera and species that may initiate the disease process.^161^ Thus, alterations in oral microbial communities and predominant bacterial genera may result in the acquisition of a “dangerous microbial ecology” early in immune system development when children and adolescents have metabolic disorders.^30^ Therefore, we may have underestimated the long‐term effects of gingivitis in children and adolescents. It can be expected that in obese and even overweight children and adolescents, these altered systemic responses may be reflected in gingival tissue early in life and “seed” the long‐term risk of periodontal tissue destruction. Thus, initiating oral bacterial translocation in response to systemic challenges at a younger age may have long‐term consequences.^165^ These chronic oral infections in children, combined with obesity and altered general health status, may have a significant cumulative impact on the risk of cardiovascular disease, diabetes onset, and other chronic inflammatory diseases.

## AUTHOR CONTRIBUTIONS

**Yunyan Zhang:** Conceptualization (lead); writing‐original draft (lead). **Tong‐Chuan He:** Conceptualization (supporting); writing‐review and editing (equal). **Hongmei Zhang:** Conceptualization (supporting); writing‐review and editing (equal).

## CONFLICT OF INTEREST STATEMENT

The authors declare no competing conflicts of interests. Tong‐Chuan He is one of the Editors‐in‐Chief of Pediatric Discovery. To minimize bias, he was excluded from all editorial decision making related to the acceptance of this article for publication.

## ETHICS STATEMENT

Not applicable.

## Data Availability

The data that support the findings of this study are available from the corresponding author upon reasonable request.

## References

[1] Kinane DF , Stathopoulou PG , Papapanou PN . Periodontal diseases. Nat Rev Dis Prim. 2017;3(1):17038. 28805207 10.1038/nrdp.2017.38

[2] Hajishengallis G , Chavakis T . Local and systemic mechanisms linking periodontal disease and inflammatory comorbidities. Nat Rev Immunol. 2021;21(7):426‐440. 33510490 10.1038/s41577-020-00488-6 PMC7841384

[3] Aldridge JP , Lester V , Watts TL , Collins A , Viberti G , Wilson R . Single‐blind studies of the effects of improved periodontal health on metabolic control in type 1 diabetes mellitus. J Clin Periodontol. 1995;22(4):271‐275. 7622632 10.1111/j.1600-051x.1995.tb00147.x

[4] Preshaw PM , Alba AL , Herrera D , et al. Periodontitis and diabetes: a two‐way relationship. Diabetologia. 2012;55(1):21‐31. 22057194 10.1007/s00125-011-2342-y PMC3228943

[5] Wang C‐WJ , McCauley Laurie K . Osteoporosis and periodontitis. Curr Osteoporos Rep. 2016;14(6):284‐291. 27696284 10.1007/s11914-016-0330-3 PMC5654540

[6] Genco RJ . Current view of risk factors for periodontal diseases. J Periodontol. 1996;67(10):1041‐1049. 10.1902/jop.1996.67.10.1041 8910821

[7] Dai W , Liu X , Han S , Li X , Xu Y , Yu Y . Influence of adipose tissue immune dysfunction on childhood obesity. Cytokine Growth Factor Rev. 2022;65:27‐38. 35595599 10.1016/j.cytogfr.2022.04.008

[8] Jacobs David R , Woo Jessica G , Sinaiko Alan R , et al. Childhood cardiovascular risk factors and adult cardiovascular events. N Engl J Med. 2022;386(20):1877‐1888. 35373933 10.1056/NEJMoa2109191 PMC9563825

[9] Soskolne AW , Bimstein E . Histomorphological study of the shedding process of human deciduous teeth at various chronological ages. Archs Oral Biol. 1977;22(5):331‐335. 10.1016/0003-9969(77)90032-2 270329

[10] Bimstein E , Eidelman E . Morphological changes in the attached and keratinized gingiva and gingival sulcus in the mixed dentition period. A 5‐year longitudinal study. J Clin Periodontol. 1988;15(3):175‐179. 3162463 10.1111/j.1600-051x.1988.tb01565.x

[11] Matsson L . Factors influencing the susceptibility to gingivitis during childhood‐a review. Int J Paediatr Dent. 1993;3:119‐127. 8260459 10.1111/j.1365-263x.1993.tb00067.x

[12] Peretz B , Machtei EM , Bimstein E . Periodontal status in childhood and early adolescence: three year follow up. J Clin Pediatr Dent. 1996;20:226‐232. 8634211

[13] Bailit HL , Baldwin DC , Hunt EE . The increasing prevalence of gingival *Bacteroides melaninogenicus* with age in children. Archs Oral Biol. 1964;9(4):435‐438. 10.1016/0003-9969(64)90028-7 14179051

[14] Kelstrup J . The incidence of *bacteroides melaninogenicus* inhuman gingival sulci, and its prevalence in the oral cavity at different ages. Periodontics. 1966;4:14‐18. 5323555

[15] Socransky SS , Manganiello SD . The oral microbiota of man from birth to senility. J Periodontol. 1971;42(8):485‐496. 4998039 10.1902/jop.1971.42.8.485

[16] Nakagawa S , Tonogi N , Kubo S , Machida Y , Okuda K , Takazoe I . Subgingival microflora in children of early childhood, school age and circumpuberty. The proportion and frequency of gram‐negative bacteria in periodontally healthy and gingivitis groups. Shoni Shikagaku Zasshi. 1991;29:72‐85. 1784868

[17] Katharina SA , Hollander Georg A , Andrew M . Evolution of the immune system in humans from infancy to old age. Proc Biol Sci. 2015;282:20143085. 26702035 10.1098/rspb.2014.3085 PMC4707740

[18] Bimstein E . Periodontal health and disease in children and adolescents. Pediatr Clin. 1991;38(5):1183‐1207. 10.1016/s0031-3955(16)38194-9 1886742

[19] Harokopakis‐Hajishengallis E . Physiologic root resorption in primary teeth: molecular and histological events. J Oral Sci. 2007;49:1‐12. 17429176 10.2334/josnusd.49.1

[20] Oh T‐J , Eber R , Wang H‐L . Periodontal diseases in the child and adolescent. J Clin Periodontol. 2002;29(5):400‐410. 12060422 10.1034/j.1600-051x.2002.290504.x

[21] Pinkham JR , Casamassimo PS , Fields HW , McTigue DJ , Nowak A . Pediatric Dentistry. Elsevier Saunders; 2005.

[22] Bimstein E , Matsson L . Growth and development considerations in the diagnosis of gingivitis and periodontitis in children. Pediatr Dent. 1999;21:186‐191. 10355010

[23] Bimstein E , Soskolne AW . A radiographic study of interproximal alveolar bone crest between the primary molars in children. ASDC J Dent Child. 1988;55:348‐350. 3170873

[24] Bishara SE . Facial and dental changes in adolescents and their clinical implications. Angle Orthod. 2000;70:471‐483. 11138651 10.1043/0003-3219(2000)070<0471:FADCIA>2.0.CO;2

[25] Sjödin B , Matsson L . Marginal bone level in the normal primary dentition. J Clin Periodontol. 1992;19(9):672‐678. 1430295 10.1111/j.1600-051x.1992.tb01717.x

[26] Peres Marco A , Macpherson Lorna MD , Weyant Robert J , et al. Oral diseases: a global public health challenge. Lancet. 2019;394(10194):249‐260. 31327369 10.1016/S0140-6736(19)31146-8

[27] Broadbent Jonathan M , Murray TW , Boyens John V , Poulton R . Dental plaque and oral health during the first 32 years of life. J Am Dent Assoc. 2011;142(4):415‐426. 21454848 10.14219/jada.archive.2011.0197

[28] D’Mello GI . Long‐term Oral and General Health Outcomes in Adolescents Who Had Extensive Decay in Early Childhood. Doctor of Clinical Dentistry Thesis. University of Otago; 2011.

[29] Armitage G . Development of a classification system for periodontal diseases and conditions. Ann Periodontol. 1999;4:1‐6. 10863370 10.1902/annals.1999.4.1.1

[30] Bimstein E , Huja PE , Ebersole Jeffrey L . The potential lifespan impact of gingivitis and periodontitis in children. J Clin Pediatr Dent. 2013;38(2):95‐99. 24683769 10.17796/jcpd.38.2.j525742137780336

[31] Saxén L . Juvenile periodontitis. J Clin Periodontol. 1980;7:1‐19. 6988466 10.1111/j.1600-051x.1980.tb01944.x

[32] Walker JD , MacKenzie IE . Periodontal diseases in children and adolescents. In: Stewart RE , Barber TK , Troutman KC , et al., eds. Dentistry. Scientific Foundations and Clinical Practice. CV Mosby Company; 1982:62.

[33] American Academy of Periodontology‐Research . Science and therapy committee, periodontal diseases of children and adolescents. Pediatr Dent. 2008;30:240‐247. 19216430

[34] Albandar JM , Brown LJ , Brunelle JA , Loe H . Gingival state and dental calculus in early‐onset periodontitis. J Periodontol. 1996;67(10):953‐959. 8910833 10.1902/jop.1996.67.10.953

[35] Albandar JM , Muranga MB , Rams TE . Prevalence of aggressive periodontitis in school attendees in Uganda. J Clin Periodontol. 2002;29(9):823‐831. 12423295 10.1034/j.1600-051x.2002.290906.x

[36] Gjermo P , Rosing CK , Susin C , Oppermann R . Periodontal diseases in central and South America. Periodontol 2000. 2002;29(1):70‐78. 12102703 10.1034/j.1600-0757.2001.290104.x

[37] Fi C , Wo W . Periodontal disease and systemic diseases: an overview on recent progresses. J Biol Regul Homeost Agents. 2021;35:1‐9. 33463138

[38] Hajishengallis G . Interconnection of periodontal disease and comorbidities: evidence, mechanisms, and implications. Periodontol 2000. 2022;89(1):9‐18. 35244969 10.1111/prd.12430 PMC9018559

[39] Loos Bruno G , Van Dyke Thomas E . The role of inflammation and genetics in periodontal disease. Periodontol 2000. 2020;83(1):26‐39. 32385877 10.1111/prd.12297 PMC7319430

[40] Hajishengallis G . Periodontitis: from microbial immune subversion to systemic inflammation. Nat Rev Immunol. 2015;15(1):30‐44. 25534621 10.1038/nri3785 PMC4276050

[41] Reeves AF , Rees JM , Schiff M , Hujoel P . Total body weight and waist circumference associated with chronic periodontitis among adolescents in the United States. Arch Pediatr Adolesc Med. 2006;160(9):894‐899. 16953012 10.1001/archpedi.160.9.894

[42] Zhao B , Jin C , Li L , Wang Y . Increased expression of TNF‐α occurs before the development of periodontitis among obese Chinese children: a potential marker for prediction and prevention of periodontitis. Oral Health Prev Dent. 2016;14(1):71‐75. 26525131 10.3290/j.ohpd.a35006

[43] Gregg EW , Shaw JE . Global health effects of overweight and obesity. N Engl J Med. 2017;377(1):80‐81. 28604226 10.1056/NEJMe1706095

[44] GBD 2015 Obesity Collaborators , Afshin A , Forouzanfar Mohammad H , et al. Health effects of overweight and obesity in 195 countries over 25 years. N Engl J Med. 2017;377(1):13‐27. 28604169 10.1056/NEJMoa1614362 PMC5477817

[45] Pan X‐F , Wang L , Pan A . Epidemiology and determinants of obesity in China. Lancet Diabetes Endocrinol. 2021;9(6):373‐392. 34022156 10.1016/S2213-8587(21)00045-0

[46] Lundin M , Yucel‐Lindberg T , Dahllöf G , Marcus C , Modéer T . Correlation between TNFalpha in gingival crevicular fluid and body mass index in obese subjects. Acta Odontol Scand. 2004;62(5):273‐277. 15841815 10.1080/00016350410000172

[47] Janem WF , Scannapieco FA , Sabharwal A , et al. Salivary inflammatory markers and microbiome in normoglycemic lean and obese children compared to obese children with type 2 diabetes. PLoS One. 2017;12(3):e0172647. 28253297 10.1371/journal.pone.0172647 PMC5333807

[48] Lehmann‐KalataSurdacka APA , Ciężka‐Hsiao E , Swora‐Cwynar E , Grzymisławski M . Clinical parameters of oral cavity, physical and microbiological properties of saliva in patients with obesity [in Polish]. Dent Med Probl. 2015;52(4):415‐423.

[49] Scorzetti L , Marcattili D , Pasini M , Mattei A , Marchetti E , Marzo G . Association between obesity and periodontal disease in children. Eur J Paediatr Dent. 2013;14(3):181‐184. 24295000

[50] Weng J , Zhou Z , Guo L , et al. Incidence of type 1 diabetes in China, 2010–13: population based study. BMJ. 2018;360:j5295. 29298776 10.1136/bmj.j5295 PMC5750780

[51] Diamond Project Group . Incidence and trends of childhood type 1 diabetes worldwide 1990–1999. Diabet Med. 2006;23(8):857‐866. 16911623 10.1111/j.1464-5491.2006.01925.x

[52] Patterson CC , Harjutsalo V , Rosenbauer J , et al. Trends and cyclical variation in the incidence of childhood type 1 diabetes in 26 European centres in the 25 year period 1989–2013: a multicentre prospective registration study. Diabetologia. 2019;62(3):408‐417. 30483858 10.1007/s00125-018-4763-3

[53] Gale EA . The rise of childhood type 1 diabetes in the 20th century. Diabetes. 2002;51(12):3353‐3361. 12453886 10.2337/diabetes.51.12.3353

[54] Dabelea D , Mayer‐Davis EJ , Saydah S , et al. Prevalence of type 1 and type 2 diabetes among children and adolescents from 2001 to 2009. JAMA. 2014;311(17):1778‐1786. 24794371 10.1001/jama.2014.3201 PMC4368900

[55] Fu J , Prasad HC . Changing epidemiology of metabolic syndrome and type 2 diabetes in Chinese youth. Curr Diabetes Rep. 2014;14(1):447. 10.1007/s11892-013-0447-z 24277673

[56] Shimazaki Y , Egami Y , Matsubara T , et al. Relationship between obesity and physical fitness and periodontitis. J Periodontol. 2010;81(8):1124‐1131. 20476888 10.1902/jop.2010.100017

[57] Chaffee Benjamin W , Weston Scott J . Association between chronic periodontal disease and obesity: a systematic review and meta‐analysis. J Periodontol. 2010;81(12):1708‐1724. 20722533 10.1902/jop.2010.100321 PMC3187554

[58] Kim E‐J , Jin B‐H , Bae K‐H . Periodontitis and obesity: a study of the Fourth Korean national health and nutrition examination survey. J Periodontol. 2011;82(4):533‐542. 21043799 10.1902/jop.2010.100274

[59] Jepsen S , Suvan J , Deschner J . The association of periodontal diseases with metabolic syndrome and obesity. Periodontol 2000. 2020;83(1):125‐153. 32385882 10.1111/prd.12326

[60] Timonen P , Niskanen M , Suominen‐Taipale L , Jula A , Knuuttila M , Ylöstalo P . Metabolic syndrome, periodontal infection, and dental caries. J Dent Res. 2010;89(10):1068‐1073. 20647498 10.1177/0022034510376542

[61] Morita T , Yamazaki Y , Mita A , et al. A cohort study on the association between periodontal disease and the development of metabolic syndrome. J Periodontol. 2010;81(4):512‐519. 20367094 10.1902/jop.2010.090594

[62] Ka K , Rousseau MC , Lambert M , et al. Metabolic syndrome and gingival inflammation in Caucasian children with a family history of obesity. J Clin Periodontol. 2013;40(11):986‐993. 23980866 10.1111/jcpe.12146

[63] Lalla E , Cheng B , Lal S , et al. Diabetes‐related parameters and periodontal conditions in children. J Periodontal Res. 2007;42(4):345‐349. 17559632 10.1111/j.1600-0765.2006.00955.x

[64] Recep O , Simsek Sera , Zerrin O , et al. The influence of type‐1 diabetes mellitus on dentition and oral health in children and adolescents. Yonsei Med J. 2008;49:357‐365. 18581583 10.3349/ymj.2008.49.3.357 PMC2615350

[65] Weintraub JA , Lopez Mitnik G , Dye BA . Oral diseases associated with nonalcoholic fatty liver disease in the United States. J Dent Res. 2019;98(11):1219‐1226. 31369716 10.1177/0022034519866442 PMC6755718

[66] Cornier M‐A , Dabelea D , Hernandez Teri L , et al. The metabolic syndrome. Endocr Rev. 2008;29(7):777‐822. 18971485 10.1210/er.2008-0024 PMC5393149

[67] Zimmet P , Alberti G , Kaufman F , et al. The metabolic syndrome in children and adolescents. Lancet. 2007;369(9579):2059‐2061. 17586288 10.1016/S0140-6736(07)60958-1

[68] Olza J , Gil‐Campos M , Leis R , et al. Presence of the metabolic syndrome in obese children at prepubertal age. Ann Nutr Metab. 2011;58(4):343‐350. 21996789 10.1159/000331996

[69] Reinehr T , de Sousa G , Toschke AM , Andler W . Comparison of metabolic syndrome prevalence using eight different definitions: a critical approach. Arch Dis Child. 2007;92(12):1067‐1072. 17301109 10.1136/adc.2006.104588 PMC2066078

[70] Baroncelli GI , Angiolini M , Ninni E , et al. Prevalence and pathogenesis of dental and periodontal lesions in children with X‐linked hypophosphatemic rickets. Eur J Paediatr Dent. 2006;7:61‐66. 16842025

[71] Chen Y , Yang Y‐C , Zhu B‐L , Wu C , Lin R , Zhang X . Association between periodontal disease, tooth loss and liver diseases risk. J Clin Periodontol. 2020;47(9):1053‐1063. 32621350 10.1111/jcpe.13341

[72] Lee K‐S , Lee SG , Kim E‐K , et al. Metabolic syndrome parameters in adolescents may be determinants for the future periodontal diseases. J Clin Periodontol. 2015;42(2):105‐112. 25469423 10.1111/jcpe.12338

[73] Holick Michael F . Resurrection of vitamin D deficiency and rickets. J Clin Invest. 2006;116(8):2062‐2072. 16886050 10.1172/JCI29449 PMC1523417

[74] Miller Walter L , Imel Erik A . Rickets, vitamin D, and Ca/P metabolism. Horm Res Paediatr. 2022;95(6):579‐592. 36446330 10.1159/000527011

[75] Prentice A . Nutritional rickets around the world. J Steroid Biochem Mol Biol. 2013;136:201‐206. 23220549 10.1016/j.jsbmb.2012.11.018

[76] Thacher Tom D , Fischer Philip R , Tebben Peter J , et al. Increasing incidence of nutritional rickets: a population‐based study in Olmsted County, Minnesota. Mayo Clin Proc. 2013;88(2):176‐183. 23374621 10.1016/j.mayocp.2012.10.018 PMC3612965

[77] Baroncelli Giampiero I , Toschi B , Bertelloni S . Hypophosphatemic rickets. Curr Opin Endocrinol Diabetes Obes. 2012;19(6):460‐467. 23108197 10.1097/MED.0b013e328358be97

[78] Beck‐Nielsen SS , Brock‐Jacobsen B , Gram J , Brixen K , Jensen TK . Incidence and prevalence of nutritional and hereditary rickets in southern Denmark. Eur J Endocrinol. 2009;160(3):491‐497. 19095780 10.1530/EJE-08-0818

[79] Endo I , Fukumoto S , Ozono K , et al. Nationwide survey of fibroblast growth factor 23 (FGF23)‐related hypophosphatemic diseases in Japan: prevalence, biochemical data and treatment. Endocr J. 2015;62(9):811‐816. 26135520 10.1507/endocrj.EJ15-0275

[80] Rafaelsen S , Johansson S , Raeder H , Bjerknes R . Hereditary hypophosphatemia in Norway: a retrospective population‐based study of genotypes phenotypes, and treatment complications. Eur J Endocrinol. 2016;174(2):125‐136. 26543054 10.1530/EJE-15-0515 PMC4674593

[81] Darveau Richard P . Periodontitis: a polymicrobial disruption of host homeostasis. Nat Rev Microbiol. 2010;8(7):481‐490. 20514045 10.1038/nrmicro2337

[82] Lalla E , Papapanou Panos N . Diabetes mellitus and periodontitis: a tale of two common interrelated diseases. Nat Rev Endocrinol. 2011;7(12):738‐748. 21709707 10.1038/nrendo.2011.106

[83] Stevenson David A , Carey John C , Byrne Janice LB , et al. Analysis of skeletal dysplasias in the Utah population. Am J Med Genet A. 2012;158(5):1046‐1054. 10.1002/ajmg.a.35327 22461456

[84] Rauch F , Glorieux Francis H . Osteogenesis imperfecta. Lancet. 2004;363(9418):1377‐1385. 15110498 10.1016/S0140-6736(04)16051-0

[85] Thomas Inas H , DiMeglio Linda A . Advances in the classification and treatment of osteogenesis imperfecta. Curr Osteoporos Rep. 2016;14:1‐9. 26861807 10.1007/s11914-016-0299-y

[86] Sillence DO , Senn A , Danks DM . Genetic heterogeneity in osteogenesis imperfecta. J Med Genet. 1979;16(2):101‐116. 458828 10.1136/jmg.16.2.101 PMC1012733

[87] Holick MF , Hossein‐Nezhad A , Tabatabaei F . Multiple fractures in infants who have Ehlers‐Danlos/hypermobility syndrome and or vitamin D deficiency: a case series of 72 infants whose parents were accused of child abuse and neglect. Dermatoendocrinol. 2017;9(1):e1279768. 29511428 10.1080/19381980.2017.1279768 PMC5832156

[88] Judge Daniel P , Dietz Harry C . Marfan's syndrome. Lancet. 2005;366(9501):1965‐1976. 16325700 10.1016/S0140-6736(05)67789-6 PMC1513064

[89] Alterio A , Alisi A , Liccardo D , Nobili V . Non‐alcoholic fatty liver and metabolic syndrome in children: a vicious circle. Horm Res Paediatr. 2014;82(5):283‐289. 25324136 10.1159/000365192

[90] Nobili V , Svegliati‐Baroni G , Alisi A , Miele L , Valenti L , Vajro P . A 360‐degree overview of paediatric NAFLD: recent insights. J Hepatol. 2013;58(6):1218‐1229. 23238106 10.1016/j.jhep.2012.12.003

[91] Alisi A , Manco M , Vania A , Nobili V . Pediatric nonalcoholic fatty liver disease in 2009. J Pediatr. 2009;155(4):469‐474. 19772998 10.1016/j.jpeds.2009.06.014

[92] Zambrano M , Nikitakis NG , Sanchez‐Quevedo MC , Sauk JJ , Sedano H , Rivera H . Oral and dental manifestations of vitamin D‐dependent rickets type I: report of a pediatric case. Oral Surg Oral Med Oral Pathol Oral Radiol Endod. 2003;95(6):705‐709. 12789152 10.1067/moe.2003.116

[93] Kaye EK , Chen N , Cabral HJ , Vokonas P , Garcia RI . Metabolic syndrome and periodontal disease progression in men. J Dent Res. 2016;95(7):822‐828. 27025874 10.1177/0022034516641053 PMC4914866

[94] Blasco‐Baque V , Garidou L , Pomié C , et al. Periodontitis induced by *Porphyromonas gingivalis* drives periodontal microbiota dysbiosis and insulin resistance via an impaired adaptive immune response. Gut. 2017;66(5):872‐885. 26838600 10.1136/gutjnl-2015-309897 PMC5531227

[95] Singer K , Lumeng CN . The initiation of metabolic inflammation in childhood obesity. J Clin Investig. 2017;127(1):65‐73. 28045405 10.1172/JCI88882 PMC5199687

[96] Nieman DC , Henson DA , Nehlsen‐Cannarella SL , et al. Influence of obesity on immune function. J Am Diet Assoc. 1999;99(3):294‐299. 10076580 10.1016/S0002-8223(99)00077-2

[97] Blüher M . Obesity: global epidemiology and pathogenesis. Nat Rev Endocrinol. 2019;15(5):288‐298. 30814686 10.1038/s41574-019-0176-8

[98] Zheng Y , Ley Sylvia H , Hu Frank B . Global aetiology and epidemiology of type 2 diabetes mellitus and its complications. Nat Rev Endocrinol. 2018;14:88‐98. 29219149 10.1038/nrendo.2017.151

[99] Cawley J . The economics of childhood obesity. Health Aff. 2013;29(3):364‐371. 10.1377/hlthaff.2009.0721 20194974

[100] Van den Bossche J , O'Neill LA , Menon D . Macrophage immunometabolism: where are we (going)? Trends Immunol. 2017;38(6):395‐406. 28396078 10.1016/j.it.2017.03.001

[101] Orr JS , Puglisi MJ , Ellacott KL , Lumeng CN , Wasserman DH , Hasty AH . Toll‐like receptor 4 deficiency promotes the alternative activation of adipose tissue macrophages. Diabetes. 2012;61(11):2718‐2727. 22751700 10.2337/db11-1595 PMC3478520

[102] Bruun JM , Lihn AS , Pedersen SB , Richelsen B . Monocyte chemoattractant protein‐1 release is higher in visceral than subcutaneous human adipose tissue (AT): implication of macrophages resident in the AT. J Clin Endocrinol Metab. 2005;90(4):2282‐2289. 15671098 10.1210/jc.2004-1696

[103] Feingold KR , Soued M , Staprans I , et al. Effect of tumor necrosis factor (TNF) on lipid metabolism in the diabetic rat. Evidence that inhibition of adipose tissue lipoprotein lipase activity is not required for TNF‐induced hyperlipidemia. J Clin Invest. 1989;83(4):1116‐1121. 2703526 10.1172/JCI113991 PMC303797

[104] Hotamisligil GS , Shargill NS , Spiegelman BM . Adipose expression of tumor necrosis factor‐alpha: direct role in obesity‐linked insulin resistance. Science. 1993;259(5091):87‐91. 7678183 10.1126/science.7678183

[105] Breslin WL , Johnston CA , Strohacker K , et al. Obese Mexican American children have elevated MCP‐1, TNF‐alpha, monocyte concentration, and dyslipidemia. Pediatrics. 2012;129(5):e1180‐e1186. 22473371 10.1542/peds.2011-2477

[106] Olefsky JM , Glass CK . Macrophages, inflammation, and insulin resistance. Annu Rev Physiol. 2010;72(1):219‐246. 20148674 10.1146/annurev-physiol-021909-135846

[107] Rathinam Vijay AK , Fitzgerald Katherine A . Inflammasome complexes: emerging mechanisms and effector functions. Cell. 2016;165(4):792‐800. 27153493 10.1016/j.cell.2016.03.046 PMC5503689

[108] Lamkanfi M , Dixit Vishva M . Mechanisms and functions of inflammasomes. Cell. 2014;157(5):1013‐1022. 24855941 10.1016/j.cell.2014.04.007

[109] Yang H , Youm Y‐H , Vandanmagsar B , et al. Obesity accelerates thymic aging. Blood. 2009;114(18):3803‐3812. 19721009 10.1182/blood-2009-03-213595 PMC2773495

[110] Kawai T , Autieri MV , Scalia R . Adipose tissue inflammation and metabolic dysfunction in obesity. Am J Physiol Cell Physiol. 2021;320(3):C375‐C391. 33356944 10.1152/ajpcell.00379.2020 PMC8294624

[111] Wolf Anna M , Wolf D , Rumpold H , Enrich B , Tilg H . Adiponectin induces the anti‐inflammatory cytokines IL‐10 and IL‐1RA in human leukocytes. Biochem Biophys Res Commun. 2004;323(2):630‐635. 15369797 10.1016/j.bbrc.2004.08.145

[112] Ziegler‐Heitbrock HW , Wedel A , Schraut W , et al. Tolerance to lipopolysaccharide involves mobilization of nuclear factor kappa B with predominance of p50 homodimers. J Biol Chem. 1994;269(25):17001‐17004. 7516328

[113] de Candia P , Prattichizzo F , Garavelli S , Alviggi C , La Cava A , Matarese G . The pleiotropic roles of leptin in metabolism, immunity, and cancer. J Exp Med. 2021;218(5):e20191593. 33857282 10.1084/jem.20191593 PMC8056770

[114] Papathanassoglou E , El‐Haschimi K , Li Xian C , Matarese G , Strom T , Mantzoros C . Leptin receptor expression and signaling in lymphocytes: kinetics during lymphocyte activation, role in lymphocyte survival, and response to high fat diet in mice. J Immunol. 2006;176(12):7745‐7752. 16751422 10.4049/jimmunol.176.12.7745

[115] Loffreda S , Yang SQ , Lin HZ , et al. Leptin regulates proinflammatory immune responses. FASEB J. 1998;12(1):57‐65. 9438411

[116] Caldefie‐Chezet F , Poulin A , Vasson MP . Leptin regulates functional capacities of polymorphonuclear neutrophils. Free Radic Res. 2003;37(8):809‐814. 14567439 10.1080/1071576031000097526

[117] Zhao Y , Sun R , You L , Gao C , Tian Z . Expression of leptin receptors and response to leptin stimulation of human natural killer cell lines. Biochem Biophys Res Commun. 2003;300(2):247‐252. 12504075 10.1016/s0006-291x(02)02838-3

[118] Karlsson EA , Sheridan PA , Beck MA . Diet‐induced obesity impairs the T cell memory response to influenza virus infection. J Immunol. 2010;184(6):3127‐3133. 20173021 10.4049/jimmunol.0903220

[119] Suriano F , Vieira‐Silva S , Falony G , et al. Novel insights into the genetically obese (*ob*/*ob*) and diabetic (*db*/*db*) mice: two sides of the same coin. Microbiome. 2021;9(1):147. 34183063 10.1186/s40168-021-01097-8 PMC8240277

[120] Ilonen J , Lempainen J , Veijola R . The heterogeneous pathogenesis of type 1 diabetes mellitus. Nat Rev Endocrinol. 2019;15(11):635‐650. 31534209 10.1038/s41574-019-0254-y

[121] Salvi GE , Collins JG , Yalda B , Arnold RR , Lang NP , Offenbacher S . Monocytic TNF alpha secretion patterns in IDDM patients with periodontal diseases. J Clin Periodontol. 1997;24(1):8‐16. 9049792 10.1111/j.1600-051x.1997.tb01178.x

[122] Karima M , Kantarci A , Ohira T , et al. Enhanced superoxide release and elevated protein kinase C activity in neutrophils from diabetic patients: association with periodontitis. J Leukoc Biol. 2005;78(4):862‐870. 16081595 10.1189/jlb.1004583 PMC1249507

[123] Ighbariya A , Weiss R . Insulin resistance, prediabetes, metabolic syndrome: what should every pediatrician know? J Clin Res Pediatr Endocrinol. 2017;9:49‐57. 29280741 10.4274/jcrpe.2017.S005 PMC5790325

[124] Kotnik P , Fischer PP , Wabitsch M . Endocrine and metabolic effects of adipose tissue in children and adolescents. Zdr Varst. 2015;54(2):131‐138. 27646920 10.1515/sjph-2015-0020 PMC4820166

[125] Hand Timothy W , Vujkovic‐Cvijin I , Ridaura Vanessa K , Belkaid Y . Linking the microbiota, chronic disease, and the immune system. Trends Endocrinol Metabol. 2016;27(12):831‐843. 10.1016/j.tem.2016.08.003 PMC5116263 27623245

[126] Esser N , Legrand‐Poels S , Piette J , Scheen AJ , Paquot N . Inflammation as a link between obesity, metabolic syndrome and type 2 diabetes. Diabetes Res Clin Pract. 2014;105:141‐150. 24798950 10.1016/j.diabres.2014.04.006

[127] Desta T , Li J , Chino T , Graves D . Altered fibroblast proliferation and apoptosis in diabetic gingival wounds. J Dent Res. 2010;89(6):609‐614. 20354230 10.1177/0022034510362960 PMC3318033

[128] Gao L , Xu T , Huang G , Jiang S , Gu Y , Chen F . Oral microbiomes: more and more importance in oral cavity and whole body. Protein Cell. 2018;9(5):488‐500. 29736705 10.1007/s13238-018-0548-1 PMC5960472

[129] Feres M , Teles F , Teles R , Figueiredo LC , Faveri M . The subgingival periodontal microbiota of the aging mouth. Periodontol 2000. 2016;72(1):30‐53. 27501490 10.1111/prd.12136 PMC5141605

[130] Hotamisligil Gökhan S . Inflammation and metabolic disorders. Nature. 2006;444(7121):860‐867. 17167474 10.1038/nature05485

[131] Socransky SS , Haffajee AD , Cugini MA , Smith C , Kent RL . Microbial complexes in subgingival plaque. J Clin Periodontol. 1998;25(2):134‐144. 9495612 10.1111/j.1600-051x.1998.tb02419.x

[132] Socransky Sigmund S , Haffajee Anne D . Periodontal microbial ecology. Periodontol 2000. 2005;38(1):135‐187. 15853940 10.1111/j.1600-0757.2005.00107.x

[133] Jensen Emilija D , Selway Caitlin A , Allen G , et al. Early markers of periodontal disease and altered oral microbiota are associated with glycemic control in children with type 1 diabetes. Pediatr Diabetes. 2021;22(3):474‐481. 33398933 10.1111/pedi.13170

[134] Mashimo PA , Yamamoto Y , Slots J , Park BH , Genco RJ . The periodontal microflora of juvenile diabetics: culture, immunofluorescence, and serum antibody studies. J Periodontol. 1983;54(7):420‐430. 6350557 10.1902/jop.1983.54.7.420

[135] Campus G , Salem A , Uzzau S , Baldoni E , Tonolo G . Diabetes and periodontal disease: a case‐control study. J Periodontol. 2005;76(3):418‐425. 15857077 10.1902/jop.2005.76.3.418

[136] da Cruz GA , de Toledo S , Sallum EA , et al. Clinical and laboratory evaluations of non‐surgical periodontal treatment in subjects with diabetes mellitus. J Periodontol. 2008;79(7):1150‐1157. 18597596 10.1902/jop.2008.070503

[137] Ganesan SM , Joshi V , Fellows M , et al. A tale of two risks: smoking, diabetes and the subgingival microbiome. ISME J. 2017;11(9):2075‐2089. 28534880 10.1038/ismej.2017.73 PMC5563960

[138] Khader Y , Khassawneh B , Obeidat B , et al. Periodontal status of patients with metabolic syndrome compared to those without metabolic syndrome. J Periodontol. 2008;79(11):2048‐2053. 18980512 10.1902/jop.2008.080022

[139] Pirih Flavia Q , Monajemzadeh S , Singh N , et al. Association between metabolic syndrome and periodontitis: the role of lipids, inflammatory cytokines, altered host response, and the microbiome. Periodontol 2000. 2021;87(87):50‐75. 34463996 10.1111/prd.12379 PMC8457155

[140] Vallianou N , Christodoulatos GS , Karampela I , et al. Understanding the role of the gut microbiome and microbial metabolites in non‐alcoholic fatty liver disease: current evidence and perspectives. Biomolecules. 2021;12(1):56. 35053205 10.3390/biom12010056 PMC8774162

[141] Albillos A , de Gottardi A , Rescigno M . The gut‐liver axis in liver disease: pathophysiological basis for therapy. J Hepatol. 2020;72(3):558‐577. 31622696 10.1016/j.jhep.2019.10.003

[142] Aron‐Wisnewsky J , Vigliotti C , Witjes J , et al. Gut microbiota and human NAFLD: disentangling microbial signatures from metabolic disorders. Nat Rev Gastroenterol Hepatol. 2020;17(5):279‐297. 32152478 10.1038/s41575-020-0269-9

[143] Kozarov Emil V , Dorn Brian R , Shelburne Charles E , Dunn WA , Progulske‐Fox A . Human atherosclerotic plaque contains viable invasive *Actinobacillus actinomycetemcomitans* and *Porphyromonas gingivalis* . Arterioscler Thromb Vasc Biol. 2005;25(3):e17‐e18. 15662025 10.1161/01.ATV.0000155018.67835.1a

[144] Kholy KE , Genco Robert J , Van Dyke TE . Oral infections and cardiovascular disease. Trends Endocrinol Metabol. 2015;26(6):315‐321. 10.1016/j.tem.2015.03.001 25892452

[145] Jain A , Batista Eraldo L , Serhan C , Stahl GL , Van Dyke TE . Role for periodontitis in the progression of lipid deposition in an animal model. Infect Immun. 2003;71(10):6012‐6018. 14500522 10.1128/IAI.71.10.6012-6018.2003 PMC201045

[146] Hayashi C , Viereck J , Hua N , et al. *Porphyromonas gingivalis* accelerates inflammatory atherosclerosis in the innominate artery of ApoE deficient mice. Atherosclerosis. 2011;215(1):52‐59. 21251656 10.1016/j.atherosclerosis.2010.12.009 PMC3057233

[147] Miyamoto T , Yumoto H , Takahashi Y , Davey M , Gibson FC , Genco CA . Pathogen‐accelerated atherosclerosis occurs early after exposure and can be prevented via immunization. Infect Immun. 2006;74(2):1376‐1380. 16428788 10.1128/IAI.74.2.1376-1380.2006 PMC1360301

[148] Graves DT , Corrêa JD , Silva TA . The oral microbiota is modified by systemic diseases. J Dent Res. 2019;98(2):148‐156. 30359170 10.1177/0022034518805739 PMC6761737

[149] De Luca F , Shoenfeld Y . The microbiome in autoimmune diseases. Clin Exp Immunol. 2019;195(1):74‐85. 29920643 10.1111/cei.13158 PMC6300652

[150] Carrizales‐Sepúlveda EF , Ordaz‐Farías A , Vera‐Pineda R , Flores‐Ramírez R . Periodontal disease, systemic inflammation and the risk of cardiovascular disease. Heart Lung Circ. 2018;27(11):1327‐1334. 29903685 10.1016/j.hlc.2018.05.102

[151] Bartova J , Sommerova P , Lyuya‐Mi Y , et al. Periodontitis as a risk factor of atherosclerosis. J Immunol Res. 2014;2014:636893‐636899. 24741613 10.1155/2014/636893 PMC3987959

[152] Chaves IdeM , Zicker MC , Laranjeira AdeO , et al. Dysbiotic oral microbiota contributes to alveolar bone loss associated with obesity in mice. J Appl Oral Sci. 2022;30:e20220238. 36417595 10.1590/1678-7757-2022-0238 PMC9724496

[153] Botero JE , Rodriguez C , Agudelo‐Suarez AA . Periodontal treatment and glycaemic control in patients with diabetes and periodontitis: an umbrella review. Aust Dent J. 2016;61(2):134‐148. 26815303 10.1111/adj.12413

[154] Scheithauer Torsten PM , Rampanelli E , Nieuwdorp M , et al. Gut microbiota as a trigger for metabolic inflammation in obesity and type 2 diabetes. Front Immunol. 2020;11:571731. 33178196 10.3389/fimmu.2020.571731 PMC7596417

[155] Simpson TC , Weldon JC , Worthington HV , et al. Treatment of periodontal disease for glycaemic control in people with diabetes mellitus. Cochrane Database Syst Rev. 2015;11(3):CD004714. 10.1002/14651858.CD004714.pub3 PMC6486035 26545069

[156] Faggion CM, Jr , Cullinan MP , Atieh M . An overview of systematic reviews on the effectiveness of periodontal treatment to improve glycaemic control. J Periodontal Res. 2016;51(6):716‐725. 26913689 10.1111/jre.12358

[157] Wang TF , Jen IA , Chou C , Lei YP . Effects of periodontal therapy on metabolic control in patients with type 2 diabetes mellitus and periodontal disease: a meta‐analysis. Medicine. 2014;93(28):e292. 25526470 10.1097/MD.0000000000000292 PMC4603101

[158] Di Paola R , Mazzon E , Maiere D , et al. Rosiglitazone reduces the evolution of experimental periodontitis in the rat. J Dent Res. 2006;85(2):156‐161. 16434734 10.1177/154405910608500208

[159] Hassumi MY , Silva‐Filho VJ , Campos‐Junior JC , et al. PPAR‐gamma agonist rosiglitazone prevents inflammatory periodontal bone loss by inhibiting osteoclastogenesis. Int Immunopharm. 2009;9(10):1150‐1158. 10.1016/j.intimp.2009.06.002 19508902

[160] Mackler SB , Crawford JJ . Plaque development and gingivitis in the primary dentition. J Periodontol. 1973;44(1):18‐24. 4509109 10.1902/jop.1973.44.1.18

[161] Matsson L . Development of gingivitis in the preschool children and young adults. J Clin Periodontol. 1978;5(1):24‐34. 353084 10.1111/j.1600-051x.1978.tb01903.x

[162] Bimstein E , Matsson L , Soskolne AW , Lustmann J . Histologic characteristics of the gingiva associated with the primary and permanent teeth of children. Pediatr Dent. 1994;3:206‐210. 8058545

[163] Tonetti MS , Mombelli A . Early‐onset periodontitis. Ann Periodontol. 1999;4(1):39‐52. 10863374 10.1902/annals.1999.4.1.39

[164] Watanabe K . Prepubertal periodontitis: a review of diagnostic criteria, pathogenesis, and differential diagnosis. J Periodontal Res. 1990;25(1):31‐48. 2137170 10.1111/j.1600-0765.1990.tb01205.x

[165] Burcham Zachary M , Garneau Nicole L , Comstock Sarah S , et al. Patterns of oral microbiota diversity in adults and children: a crowdsourced population study. Sci Rep. 2020;10(1):2133. 32034250 10.1038/s41598-020-59016-0 PMC7005749

